# Surgical jejunostomy and radiological gastro-jejunostomy tube feeding in children: risks, benefits and nutritional outcomes

**DOI:** 10.1007/s00383-018-4303-8

**Published:** 2018-07-16

**Authors:** Rashmi R. Singh, Simon Eaton, Derek J. Roebuck, Alex M. Barnacle, Samantha Chippington, Kate M. K. Cross, Paolo De Coppi, Joe I. Curry

**Affiliations:** 1 Department of Paediatric Surgery, UCL Great Ormond Street Institute of Child Health and Great Ormond Street Hospital for Children, London, UK; 2 Department of Radiology, UCL Great Ormond Street Institute of Child Health and Great Ormond Street Hospital for Children, London, UK

**Keywords:** Jejunostomy, Gastro-jejunostomy tube, Nutritional outcome

## Abstract

**Purpose:**

Radiologically inserted gastrojejunal tubes (RGJ) and surgical jejunostomy (SJ) are established modes of jejunal feeding. The aim of the study is to review nutritional outcomes, complications and the practical consideration to enable patients and carers to make informed choice.

**Methods:**

Retrospective review of patient notes with a RGJ or SJ in 2010, with detailed follow-up and review of the literature.

**Results:**

Both RGJ and SJ are reliable modes to provide stable enteral nutrition. Both have complications and their own associated limitations.

**Conclusions:**

The choice has to be tailored to the individual patient, the social care available, the inherent medical disease and risk/benefit of repeated anaesthetic and radiation exposure. RGJ and SJ are important tools for nutritional management that achieve and maintain growth in a complex group of children. The risk and benefits should be reviewed for each individual patient.

## Introduction

In children with gastrointestinal dysfunction, jejunal access can be used for enteral feeding. The inability to tolerate gastric feeding can be due to a variety of reasons including, gastro-oesophageal reflux, gastric dysmotility and poor gastric compliance [1]. Historically, these children received a surgical jejunostomy (SJ). Radiologically inserted gastrojejunal tubes (RGJ) are now more commonly used than surgical jejunostomy [2, 3]. Our aim was to understand whether this change in practice provides additional benefit for the patient/family or institution.

We reviewed outcomes in children with surgical feeding jejunostomy and radiologically inserted trans-gastric jejunal feeding tubes at our institute.

## Methods

After appropriate institutional audit approval (no. 1035), a retrospective review to identify patients who had a jejunostomy in the year 2010 was performed. At the commencement of the study, we wanted to have children with at least 3 years of follow-up. We reviewed the hospital coding database and identified seventy-eight children who had a new jejunostomy in 2010. Of these, 29 children were excluded as detailed note review revealed that they either did not have the primary jejunostomy in 2010 or the jejunostomy was a part of laparotomy to act as a de-functioning stoma. Data were extracted on outcomes from those with a ‘de novo’ RGJ or SJ from clinic and discharge letters, admission records, imaging procedures and inpatient stay records. Procedures were performed by consultant interventional radiologists or paediatric surgeons, or by trainees at the specialist registrar level under direct supervision of a consultant. In our institute, the RGJ and SJ devices used are approved (CE marked) and marketed in the UK and EU but is not FDA approved. The RGJ had initial 9-Fr Freka gastrostomy devices converted to 15-Fr under general anaesthesia. Thereafter, 9-Fr coaxial jejunal extension was inserted under fluoroscopic guidance. This was done by pushing the extension tube (loaded with its stiffening wire) through the pylorus and then advancing it over the wire until it reached the jejunum. If this was not possible a shaped angiographic catheter and guidewire was used to access the jejunum, and the jejunal catheter advanced over the guidewire. Balloon gastrostomy tubes were removed (without general anaesthesia) and a shaped angiographic catheter and guidewire were used to access the jejunum through the gastrostomy. A balloon GJ device Kimberley Clark or AMT was then inserted over the guidewire [4].

We collected data for RGJ and SJ patients with respect to demography, neurological diagnosis, indication, previous anti-reflux surgery, complications, hospital admission, further surgery, removal of the device and follow-up. We reviewed their weights before and after RGJ or SJ as an outcome measure. Weight-for-age *Z* scores (Standard deviation scores) were calculated using the LMS (Lambda–Mu–Sigma) growth add-in [5] for Microsoft Excel 2010 (Microsoft Corporation) program, using British 1990 reference data [6]. A *Z* score of 0 is equivalent to 50th centile, − 1 to 16th centile and − 2 to 2nd centile. Malnourished children were defined as weight *Z* score of − 2 or less. Growth over time was assessed as mean change in *Z* score per year by Multilevel modelling using MlWin 2.36 (Centre for Multilevel Modelling, University of Bristol, Bristol, UK).

## Results

Forty-eight children had a jejunostomy for feeding in the year 2010 and they were included in the study. In the RGJ group, thirty-one children had pre-existing gastrostomy, while 5 had a new Freka gastrostomy inserted through which the jejunal extension was passed (under fluoroscopic guidance). Demographic data are presented in Table 1. More than half of the patients who received either RGJ or SJ were neurologically impaired (58% and 67% respectively). Indications for jejunal feeding are listed in Table 2. In 83% of SJ and 69% of RGJ, it was recurrent gastro-oesophageal reflux (GOR). This was confirmed by either a contrast study or pH study. The majority of children in each group had a previous anti-reflux operation, *n* = 19/36 (53%) in RGJ and *n* = 6/12 (50%) in SJ (Table 3).

**Table 1:** Demographic data of children receiving a jejunostomy in 2010

	RGJ (*n* = 36)	SJ (*n* = 12)
Age median (range) in months	37 (5–202)	41 (6–213)
Sex	17 males/19 females	11 males/1 female
Neurological impairment	21 (58%)	8 (67%)
Other indications—gastric dysmotility associated with	Metabolic disorder 3	Oesophageal atresia 1
	Oesophageal atresia 2	Gut failure due to immune dysregulation 1
	Failure to thrive 2	Metabolic disorder 1
	Cardiac disorder 2	Endocrine disorder 1
	Malignant disorder 2	
	Endocrine disorder 1	
	Dystrophic epidermolysis bullosa 1	
	Short bowel syndrome after resection for multiple atresia 1	
	Severe combined immune deficiency 1	

**Table 2:** Indications for jejunal feeding

	RGJ (*n* = 36)	SJ (*n* = 12)
Recurrent GOR	25 (69%)	10 (83%)
Not tolerating gastric feeds	11 (31%)	0
Duodenal obstruction due to: Multiple intestinal strictures	0	1 (8%)
Long-gap oesophageal atresia: gastric pull-up + SJ	0	1 (8%)

*GOR* gastro-oesophageal reflux

**Table 3:** Previous surgery and further surgery after a RGJ or SJ

	RGJ (*n* = 36)	SJ (*n* = 12)
Previous surgery *n* (%)	Fundoplication + G 15 (42)	Fundoplication + G 5 (42)
	Gastrostomy 12 (33)	Gastrojejunostomy 4 (33)
	Fundoplication + revision + G 4 (11)	Laparotomy 3 (25)
	None 5 (14)	Gastrostomy 3 (25)
		Fundoplication + revision twice + G 1 (8)
		None 1 (8)
		(*n* = 4 had more than 1 procedure)
Further surgery *n* (%)	Revision fundoplication + G 6 (17)	Re-fashioning 2 (17)
	SJ 4 (11)	Laparotomy (bowel obstruction) 2 (17)
	Removal of buried bumper 3 (8)	
	Fundoplication + G 2 (6)	
	Revision of G 3 (8)	
	(*n* = 3 had more than 1 procedure)	

*G* gastrostomy

The type of surgical jejunostomy depended on the choice of the operating surgeon and the disease aetiology. Most of the surgeons preferred formation of Roux-en-Y jejunostomy (*n* = 7), the others were Witzel tunnel (*n* = 4) and laparoscopy assisted (*n* = 1). The initial tube was a Malecot 14-Fr catheter that was converted to a balloon button gastrostomy at 6 weeks. The Witzel tunnel was performed over a duodenal feeding tube of size ranging from 6 to 9 Fr.

There were four major complications in each of the RGJ (11%) and SJ (33%) groups. Four buried bumpers in RGJ, while two cases of bowel obstruction (one due to colonic volvulus and ventral hernia at fundoplication site in a Roux-en-Y SJ, and the other intussusception with small bowel volvulus in a Witzel tunnel SJ) and two cases of stenosis/atresia at skin level needing re-fashioning of SJ.

The children who received a SJ had various rare disease pathologies. One child had multiple intestinal abscesses as a part of global immune deficiency. He had multiple resections and anastomoses of the small bowel during formation of SJ. There was stenosis of the jejunostomy later as a part of the disease process and it required revision. Another child with congenital myopathy and oesophageal stricture developed stenosis of the stoma mouth and needed re-fashioning of the jejunostomy.

The RGJ group needed tube replacement at a median of 1.3 (0–20) times/year. Balloon RGJ devices every 4–6 months, and the 15-Fr/9-Fr Freka system every 1–2 years as a planned procedure. Fifteen children needed further 18 operations (Table 3). In 20/36 children, RGJ was removed after 0.8 years (0.1–2.4). Four were fed orally, three oral with gastrostomy, five via gastrostomy alone, five had fundoplication plus gastrostomy and three converted to SJ.

Twelve children had SJ, which was a part of laparotomy in 5/12. Four (33%) had SJ after RGJ. SJ was reversed in one orally fed child.

**Fig. 1:**
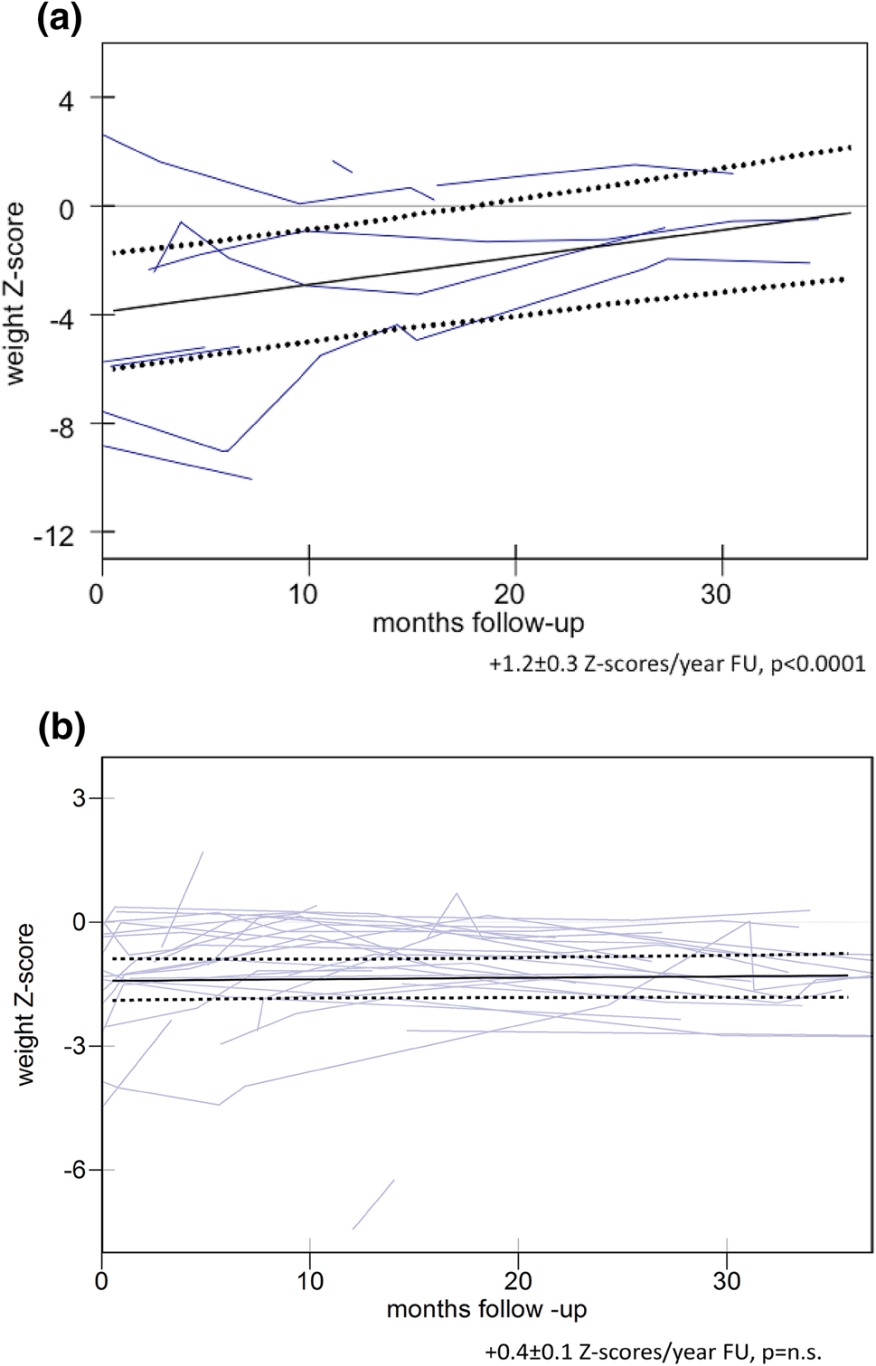
**a** Weight Z scores for children after SJ. **b** Weight Z scores for children after RGJ. Individual patients are shown together with the mean trend line with 95% confidence intervals of the mean, analysed by multilevel modelling

Nutritional outcome was measured as weight Z scores over time. RGJ children were on average slightly underweight (mean − 1.4 ± standard error 0.26 *Z* scores) at jejunostomy, with 4/36 (11%) of children malnourished (less than − 2 *Z* scores). RGJ children grew stably (+ 0.4 ± 0.1 *Z* scores per year follow-up, *p* = 0.58).SJ children were on average significantly malnourished (− 3.7 ± 0.99 *Z* scores) at the time of jejunostomy, with 4/12 (33%) of children less than − 2 *Z* scores. Growth significantly improved following SJ (+ 1.2 ± 0.3 *Z* scores/year follow-up, *p* < 0.0001) (Fig. 1).

RGJ were followed up for a median of 2.4 (0.18–3.4) years, while SJ were followed up for a median of 1.8 (0–3.5) years.

## Discussion

Complex, neurologically impaired children have a range of feeding difficulties from uncoordinated swallow to GORD and gastrointestinal dysmotility. Once maximal medical therapy has failed, management options include gastric tube feeding, anti-reflux procedure, jejunal feeding or a combination. The rate of recurrent GORD after an anti-reflux procedure is between 10 and 14% [7–9]. For these patients, a re-do fundoplication has a high failure rate of 20–30% [10, 11]. Jejunostomy feeding has been previously reported and may be preferred over a redo fundoplication [3, 12]. Long-term outcomes following SJ and RGJ have been compared in a previous series [1]. They concluded that SJ are more stable feeding access devices with fewer complications.

There is a reported association of buried bumpers and gastro-jejunal tubes [13] (when the gastric component of the tube is inserted as a percutaneous technique). This can be due to the reluctance of the carer to advance the gastro-jejunal device, for fear of dislodging the jejunal component. Due to the presence of the jejunal tube, the carers are advised to only advance the gastrostomy tube and not to rotate it. Often the jejunal component of the RGJ is routinely replaced and the gastrostomy device remains in situ for a longer duration than intended.

SJ has been reported to have a high complication rate. Williams et al. reported major complication rate of 37% [14] and Smith et al. reported a major complication rate of 31% [15] with Roux-en-Y SJ. Taylor et al. reported volvulus around Roux-en-Y SJ in 5 out of 25 patients (20% complication rate) [16]. Egnell et al. reported 33% re-operation rate after SJ for small bowel obstruction, perforation, wound rupture tube dislodgement and tube leak [17]. At our institute, the surgical technique has evolved over time, with Roux-en-Y SJ, having a shorter stem of the Roux-en-Y limb, thus minimizing the risk of volvulus.

There has been reported increased association of intussusception and jejunal perforation with RGJ in children less than 6 kg of weight and 6 months of age [18]. We did not experience this. However, for children < 10 kg, we prefer a 15-Fr/9-Fr Freka system over the thick and relatively stiff 16-Fr Kimberley Clark device.

Determining nutritional benefit from RGJ or SJ in this complex group of children is challenging. The use of weight *Z* scores before and after the jejunostomy insertion gives an objective measure of the probable effect of the intervention on nutrition. As the underlying disease progresses, becomes stable or regresses, there may be an effect on absorption of nutrients from the gut and maintenance of nutrition. Changes in *Z* scores are multi-factorial and include changes in feeding regime, formulas used, and other background general illness. The patients who had an RGJ were slightly underweight at the start and maintained stable weight gain (manifested as stable *Z* score). The patients who had a SJ, on the other hand, were significantly malnourished at SJ insertion and their growth improved significantly (significant increase in weight *Z* score). Rather than just the effect of the jejunostomy, this result reflects the fact that patients who had a SJ had the jejunostomy after progressive deterioration of the primary disease and failure of escalating nutritional interventions. Given our data, we conclude that both RGJ and SJ are effective as they have a stabilizing effect on reliable delivery of nutrition.

**Fig. 2:**
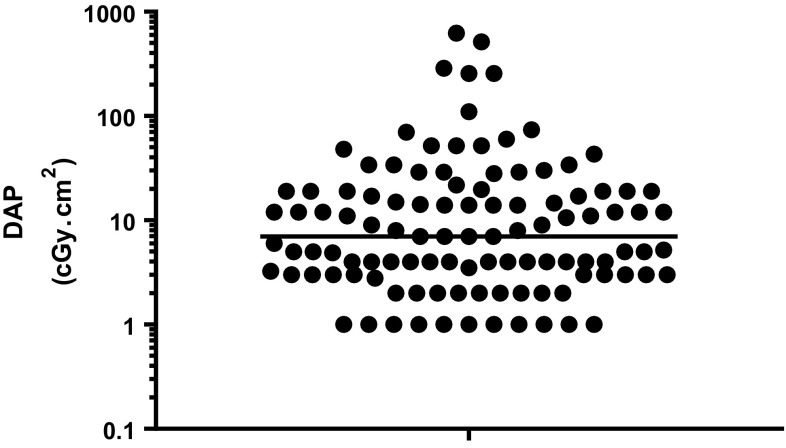
Dose–area product (DAP) for 110 children having an RGJ tube change (Horizontal line denotes the median DAP)

Another aspect that requires consideration in the choice of procedure to be offered is the radiation dose received, not just at the initial RGJ insertion, but also each time the tube is replaced. In a sample of 110 consecutive patients (not the same patients as the primary study population, as data were not available), the median radiation dose–area product (DAP) for a change of RGJ tube was 7 µGy·m^2^ (0–622 µGy·m^2^, Fig. 2) with a median fluoroscopy time of 25 s (0 s–40 min). There is no clear consensus regarding the additional cumulative lifetime risk of radiation to patients [19].

The average cost of insertion of an SJ at our institution is around £11,000 as the procedure involves a general anaesthetic, theatre time and inpatient stay of several days (although several SJ patients had SJ insertion together with another abdominal procedure), whereas the average cost associated with a day case admission and insertion of RGJ in the radiology suite is around £590 (data obtained from the hospital costings department). The recurrent costs associated with each is also likely to be different: RGJ will require changing in interventional radiology approximately every 6 months, whereas the SJ tube can be replaced in an outpatient appointment. A full cost-effectiveness analysis, including the cost of complications, however, was not undertaken as part of the current study.

In adults, laparoscopic jejunostomy has been reported in 299 patients with low rate of post-operative small bowel obstruction [20]. Laparoscopic Roux-en-Y jejunostomy has been reported in five children one of whom required dilatation for stomal stenosis [21]. Esposito et al. have described laparoscopic-assisted jejunostomy formation in ten neurologically impaired children [22]. One patient (10%) died 1 year after the procedure of unknown causes. The other complications were four (40%) peristomal hernias, two (20%) device dislocation and one peristomal granuloma.

Direct percutaneous endoscopic jejunostomy has been reported in five children with good results [23]. However, in a large series of 286 adult patients, the success rate was 68% and the procedure was associated with a complication rate of 10% [24]. Recently percutaneous laparoscopic endoscopic jejunostomy has been reported in sixteen children [25]. They had two complications (12.5%) of small bowel volvulus, which required surgical intervention.

The effect of repeated hospital admission for RGJ tube replacement with inadvertent displacement on the quality of life of the patient and caregivers has not been studied in the adult or paediatric literature. We have also become aware of some families’ difficulty in sourcing RGJ tube placement regularlywhen their child transitions to adult care. This, in our experience, has been the reason for families choosing to have an SJ over a RGJ. A formal quality of life assessment for the patient and caregivers is much needed.

Although there are papers citing increased morbidity after a RGJ [26, 27], this remains a feasible alternative in the fragile patient with compromised respiratory function due to recurrent aspiration [28].

## Conclusions

To conclude, our data suggest that both RGJ and SJ are reliable modes to provide stable enteral nutrition. The choice of the procedure and device has to be tailored to the individual patient, the social care available, the inherent medical disease and risk/benefit of repeated anaesthetic and radiation exposure.

This study is not intended to directly compare SJ and RGJ, as this group of patients represent a heterogeneous population who often have had a trial of nasogastric, gastric, naso-jejunal, RGJ feeding before becoming significantly malnourished, thus resorting to a SJ. Although RGJs require more device maintenance than SJ, they have less significant complications. RGJ can be used as a temporary stabilizing measure after failed anti-reflux operations in the neurologically impaired. Insertion or replacement through an existing gastrostomy under radiological guidance obviates the need for a general anaesthetic in most cases.

A consistently high DAP for tube changes in an individual patient might be a relative indication to convert from a RGJ strategy to SJ. The cost and inconvenience associated with tube replacement and hospital admission is another important consideration. This information should be presented to the family while counselling for the choice of jejunal tube.

RGJ and SJ are important tools for nutritional management that achieve and maintain growth in a complex group of children. The risk and benefits should be reviewed for each individual patient.
